# Hygiene as a Branch of Medical Education

**Published:** 1855

**Authors:** 


### HYGIENE AS A BRANCH OF MEDICAL EDUCATION.

1. It is a fact worthy of particular notice, that up to the present time the science and practice of medicine have been confined mainly to the investigation of the treatment of maladies; and that comparatively few attempts have been made by the professors of the healing art to comprehend those great laws, under the influence of which diseases are produced and fostered.

2. True it is, that in the literature of medicine many volumes are to be found, in which references are made to the question of the presumed cause of some special disorder or class of disorders. But such isolated remarks, though valuable in themselves, and often giving evidence of great research, have proved of but little utility in the solution of those greater problems—the determination of the elements of diseased action, the investigation of the prime causes of disease, and the discovery of means for the absolute removal of such causes.

3. The reason of this inattention to the principles of preventive medicine can be understood only by a reference to the past history of medical science. Medicine, as an occupation, commenced with the treatment of actual disease. Great triumphs were achieved by this proceeding; and, as the system went on improving from age to age, the public and the profession became so accustomed to it as a system, that to make any departure from it was little less than downright heresy and innovation. Disease and medical skill were the two objects of the public faith as regarded health. If they were to be brought into collision, the battle must be fairly arrayed for both sides. Then the struggle would come; and sometimes disease and sometimes medical skill would have the victory.

4. It is due to the firm hold of these opinions on the popular mind, that the progress of the science of preventive medicine has so long been retarded. For so convinced are the people of the propriety of keeping up routine, that men of true science in the medical profession have been, and still are, afraid of saying too much on hygienic subjects, lest the people, by whom they are supported, should stamp them as not "practical," and lest they, as restorers of life and health, should lose caste by indicating too plainly their knowledge of those conditions by which alone life and health are maintained.

5. Thus the science of hygiene or preventive medicine is as yet, in this country, in its veriest infancy. Its cultivation and application are left in the hands of a few earnest individuals, who, from the smallness of their number, are quite unable to grapple with all the difficulties and prejudices that lie in their way. Let us glance briefly at the processes by which these difficulties and prejudices may be most speedily removed.

6. In the first place, a great and wise concession must be made by the public; so that the physician, the surgeon, and the general practitioner of medicine may not be restrained from offering any useful fact or suggestion on ventilation, cleanliness, influence of temperature, and the like, by a positive conviction that the giving birth to such a fact or suggestion would almost inevitably ruin his reputation as a practitioner, without affording him compensation as a scientific discoverer.

7. In the next place, the professors of medicine must take a more extended view of their position. Setting at absolute defiance the gross and vulgar superstitions of the people on matters pertaining to disease, they must commence an internal reform; not by lessening the importance of curative medicine, or by paying less attention to it as a grand and noble study; but by shewing in their labours that there is another and a sister science, the prosecution of which leads to the prevention of human suffering, and which holds the same position between health and disease, as curative medicine holds between disease and death.

8. In endeavouring to place the principles of preventive medicine on their true basis, the first step to be taken is to teach those principles to the rising generation of medical practitioners. In every school of medicine there ought to be, and we have reason to believe there soon will be, a professor of hygiene, holding a position as important as the professor of physiology or of practical medicine. In every collegiate or university examination for licences and degrees, a knowledge of hygiene should be demanded. If these two plans were followed, the study of the principles of preventive medicine would soon be considered by the profession and the public as a legitimate and even a necessary pursuit.

9. At this time, when some thousands of Englishmen, engaged on a great service in which the national honour and prosperity are deeply concerned, have been allowed to perish outright, not by the battle strife, not by the inefficiency of the curative art, but by an unpardonable and almost savage ignorance of the first and simplest of hygienic laws; at this time, we say, with the catastrophe of the decimation of a great army before our eyes, it is surely of some moment that steps be at once taken to brush away for ever every prejudice and obstacle, that shall check the progress of a science which includes in its embrace a complete knowledge of the laws of health and life.

